# Prevalence of dysglycemia and associated risk factors in patients with pancreatic benign and low-grade malignant tumors before pancreatic surgery: A prospective observational study

**DOI:** 10.3389/fendo.2022.960843

**Published:** 2022-10-27

**Authors:** Jie Yang, Jia Zhang, Rui Wang, Ya Liu, Yonghua Chen

**Affiliations:** ^1^ Department of Pancreatic Surgery, West China Hospital of Sichuan University, Chengdu, China; ^2^ Department of Breast Surgery, West China Hospital of Sichuan University, Chengdu, China

**Keywords:** diabetes mellitus, pancreatic tumors, pancreatic endocrine function, remnant pancreatic volume, risk factors

## Abstract

**Background:**

Pancreatic benign and low-grade malignant tumors (PBLMT) have experienced a rapid increase in incidence rates worldwide. Few studies have focused on the glucose metabolism status of patients with PBLMT before pancreatic surgery.

**Methods:**

From August 2017 to June 2018, 70 patients with PBLMT were prospectively screened for abnormalities in glucose metabolism by an oral glucose tolerance test (OGTT) before pancreatic surgery. Patients were classified as having normal glucose tolerance (NGT), prediabetes mellitus (pre-DM), or new-onset DM (NOD) according to the American Diabetes Association (ADA) criteria. Glucose metabolism indices were calculated based on the OGTT parameters. Tumor volume and remnant pancreatic volume (RPV) were measured by computed tomography.

**Results:**

Forty-nine of 70 patients with PBLMT developed dysglycemia (pre-DM and NOD). RPV was smaller in the pre-DM (57.44 ± 18.20 cm^3^ vs. 70.48 ± 14.08 cm^3^, *P* = 0.001) and NOD groups (37.38 ± 20.40 cm^3^ vs. 70.48 ± 14.08 cm^3^, *P* < 0.001) than in the NGT group. The homeostasis model assessment of β-cell function (HOMA2-β), insulinogenic index (IGI), and insulin secretion/insulin resistance index (ISSI-2) were worse in the pre-DM and NOD groups compared with NGT group (all *P* < 0.05). After univariate and multivariate analyses, age over 60 years (*P* = 0.049, OR = 5.76, 95% CI: 1.01-32.92) and RPV less than 49.36 cm^3^ (*P* = 0.024, OR = 8.59, 95% CI: 1.34-55.22) were recognized as independent risk factors for dysglycemia. The analysis of all patients revealed inverse correlations between RPV and both in age (r = -0.28, *P* = 0.019) and tumor volume (r = -0.28, *P* = 0.032). Positive correlations were found between RPV and both IGI (r = 0.29, *P* = 0.019) and ISSI-2 (r = 0.39, *P* = 0.0011).

**Conclusion:**

In patients with PBLMT, 70% had dysglycemia before surgery. Old age and a reduction in RPV were independent risk factors for developing dysglycemia before pancreatic surgery. The decisions to treat PBLMT with resection should hinge more on the risk of dysglycemia as well as potential malignancy.

## Introduction

Pancreatic benign and low-grade malignant tumors (PBLMT), such as the serous or mucinous cystic tumors, intraductal papillary mucinous tumors (IPMN), solid pseudopapillary tumors, and pancreatic neuroendocrine tumors (PNET), have been acknowledged worldwide for their rapid growth in incidence rate (1, 2). Treatment of PBLMT with partial pancreatectomy or organ-preserving pancreatic surgery could preserve the pancreas parenchyma and exocrine and endocrine functions (3, 4). However, patients suffering from these pancreatic tumors might develop diabetes mellitus (DM) before and after pancreatic surgery.

Pancreatogenic DM accounts for approximately 8% of patients with diabetes in Western countries, with chronic pancreatitis and pancreatic cancer currently recognized as the first two causes (5, 6). Pancreatic tumors can alter endocrine and metabolic conditions before and after surgery (7). Research has indicated an epidemic of DM in patients with PBLMT after pancreatic surgery, albeit at a lower rate than in patients with chronic pancreatitis (8, 9). Indeed, few studies have focused on the glucose metabolism status of PBLMT patients. The prevalence of DM and impaired fasting glucose was 24.4% in PNET patients who did not receive medical treatment (10). Based on a meta-study, the prevalence of new-onset DM in IPMN patients was 6% (68 of 1,202) with a range of 1.5% to 29% (11). However, the lack of a clear definition or the use of fasting plasma glucose levels alone might not accurately evaluate the glycemic traits of PBLMT patients.

With the present study, we sought to provide prospective insight into the actual glucose metabolism condition in patients with PBLMT by an oral glucose tolerance test (OGTT) before pancreatic surgery. Furthermore, we explored the risk factors for the PBLMT patients, especially involving the tumor volume and remnant pancreatic volume.

## Methods

### Study design

**Figure 1:**
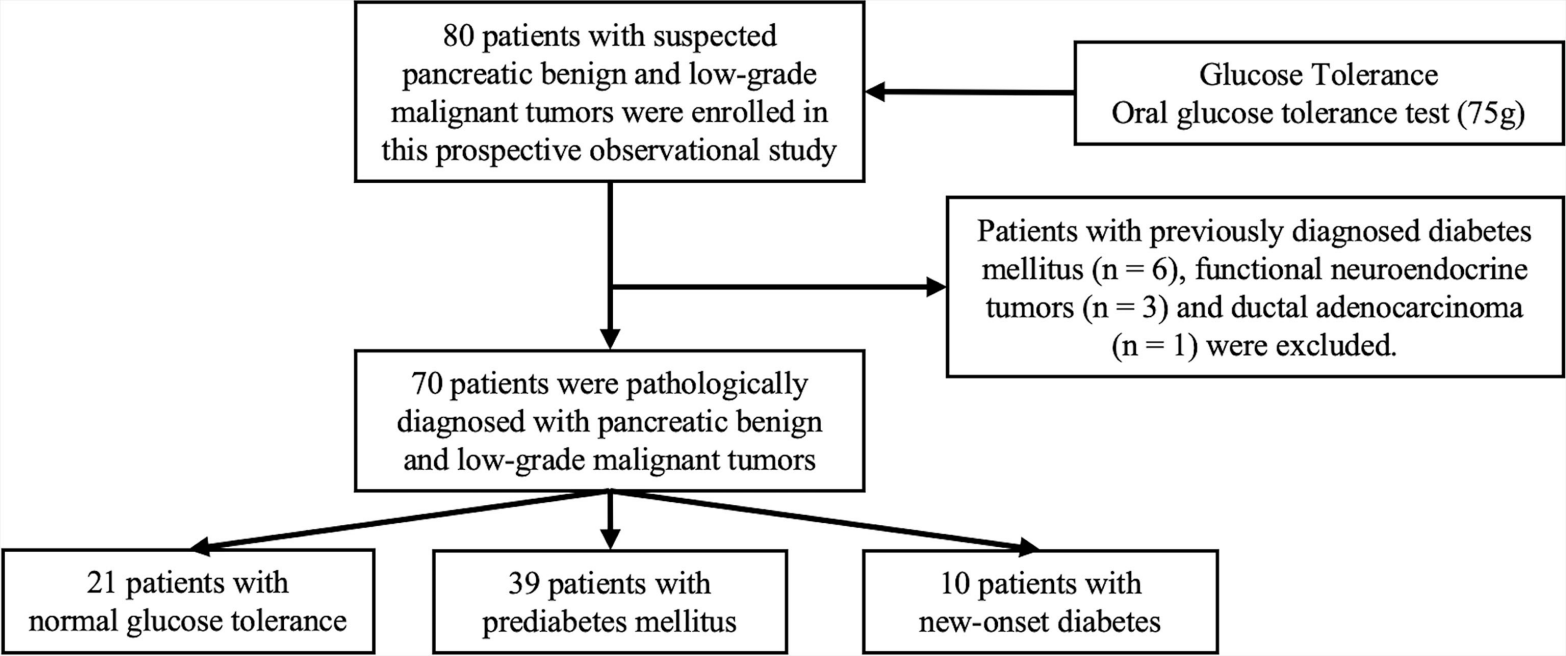
Flow chart of the study.

From August 2017 to June 2018, 80 patients with suspected PBLMT were prospectively screened for abnormalities in glucose metabolism before pancreatic surgery. We excluded six patients previously diagnosed with DM, three pathologically diagnosed with functional neuroendocrine tumors, and one with IPMN-developed ductal adenocarcinoma. The remaining 70 participants were included in the analysis (**Figure 1** and **Supplementary Table 1**). The study was approved by the West China Hospital of Sichuan University Biomedical Research Ethics Committee (2014 Trail No.37) and conformed to the Declaration of Helsinki. Informed consent was acquired from all individual participants and guardians included in the study.

### Biochemical parameters

Subjects were evaluated at West China Hospital after at least an 8-hour overnight fast before surgery. A 3-hour oral glucose tolerance test (OGTT) was performed with a 75-g glucose load. Blood samples were collected at 0, 30-, 60-, 120-, and 180-min time points, and each sample’s plasma glucose, insulin, and C-peptide concentrations were tested. Meanwhile, HbA1c was measured. Patients were classified as having normal glucose tolerance (NGT, fasting plasma glucose < 5.6 mmol/L and 2-hour plasma glucose < 7.8 mmol/L), prediabetes mellitus (pre-DM, fasting plasma glucose 5.6-6.9 mmol/L and/or 2-hour plasma glucose 7.8-11 mmol/L), or new-onset DM (NOD, fasting plasma glucose ≥ 7 mmol/L and/or 2-hour plasma glucose ≥ 11.1 mmol/L), according to the American Diabetes Association (ADA) criteria (12).

The areas under the curve of glucose (AUC_glucose_), insulin (AUC_insulin_), and C-peptide (AUC_C-peptide_) were calculated using trapezoidal integration from 0 to 180 min. The homeostasis model assessment of insulin resistance (HOMA2-IR) or β-cell function (HOMA2-β) was calculated from fasting glucose and insulin levels as previously described (13). The Matsuda index was adopted as a measure of insulin sensitivity (14). To evaluate β-cell function corrected for the degree of insulin sensitivity, two surrogate measures were adopted in this study: 1) insulinogenic index (IGI), which was calculated by the ratio of 30 min insulin minus fasting insulin to 30 min glucose minus fasting glucose (ΔInsulin30:ΔGlucose30) (15), was validated against first-phase insulin secretion on intravenous glucose tolerance testing, and 2) insulin secretion/insulin resistance index (ISSI-2, or disposition index) was calculated by IGI multiplied by the Matsuda index (15).

Carbohydrate antigen 19-9 (CA 19-9), total bilirubin, alanine transaminase, albumin, triglyceride, cholesterol, high-density lipoprotein, low-density lipoprotein, beta-hydroxybutyrate, and total bile acids were measured in the laboratory department of West China Hospital of Sichuan University according to national standards.

### Remnant pancreatic volume and tumor volume

Abdominal computed tomography (CT) scans were used to analyze the remnant pancreatic volume (RPV) as described previously (16, 17). The pancreas was identified on each image, and the outline of normal pancreatic tissue, which excluded the tumor region, was annotated by freehand region estimation to generate a pancreatic area for each slice. Pancreatic volume was calculated by multiplying the estimated area of pancreatic tissue on each image slice by the interval between slices. The tumor volume of each patient was calculated through the same procedure as RPV, and both were assessed independently by two authors (JY and JZ). RPV and tumor volume were classified by thirds of the distribution in the logistic regression analysis.

### Statistical analysis

All the data were analysed by the SPSS version 24.0 (IBM, New York, US). Data are presented as frequencies for categorical variables and mean ± standard deviation (SD) for continuous variables. Differences between groups were analysed using the independent samples t test, Mann–Whitney U nonparametric test for continuous data, and Pearson’s chi-square test for categorical data. We used univariate and multivariate logistic regression models to compute the odds ratio (OR) with a 95% confidence interval (CI) estimate of relative risk. Correlations were determined using Pearson’s correlation coefficient as appropriate. A two-sided *P* value less than 0.05 was regarded as statistically significant.

## Results

### Characteristics of patients

Of the included 70 PBLMT patients, 43 were pathologically diagnosed with pancreatic cystic tumors, 14 were diagnosed with solid pseudopapillary tumors, and 13 were nonfunctional neuroendocrine tumors. Preoperative OGTT revealed that the prevalence of NGT, pre-DM, and NOD was 30%, 55.7%, and 14.3%, respectively. **Table 1** presents the characteristics of patients with different glucose tolerance statuses. Patients with NGT were younger than pre-DM and NOD (*P* = 0.002 and *P* = 0.001, respectively). Patients in the NOD group had the highest HbA1c levels (6.65 ± 0.78%), compared with the pre-DM group (5.77 ± 0.32%, *P* = 0.015) and the NGT group (5.29 ± 0.21%, *P* = 0.002). HbA1c level was higher in the pre-DM group than in the NGT group (*P* < 0.001). The median tumor volume of all involved patients was 11.50 cm³ (range from 0.09 to 216.97 cm³). Tumor volume did not differ between NGT (24.28 ± 32.05 cm^3^) and pre-DM (22.89 ± 35.61 cm^3^) patients (*P* = 0.882); however, both were smaller than that in NOD patients (64.69 ± 74.12 cm^3^, *P* = 0.041 and *P* = 0.049, respectively). The median RPV was 58.67 cm³ (range from 7.50 to 91.93 cm³) in all patients. RPV was smaller in pre-DM (57.44 ± 18.20 cm^3^ vs. 70.48 ± 14.08 cm^3^, *P* = 0.001) and NOD (37.38 ± 20.40 cm^3^ vs. 70.48 ± 14.08 cm^3^, *P <* 0.001) patients than in NGT patients. Compared with the pre-DM group, patients with NOD still showed smaller RPVs (*P <* 0.001). There were no significant differences among groups regarding sex, body mass index (BMI), tumor location, tumor type, pancreatic main duct dilation, CA19-9, bilirubin, triglyceride, or bile acid levels.

**Table 1:** Characteristics of PBLMT patients with different glucose tolerance statuses.

Characteristics	NGT (n = 21)	pre-DM (n = 39)	NOD (n = 10)	*P* value 1	*P* value 2	*P* value 3
Sex (female)	17	22	9	0.088	0.575	0.130
Age (year)	42.1 ± 12.1	52.5 ± 11.8	58.9 ± 13.7	0.002	0.001	0.141
BMI (kg/m^2^)	22.25 ± 3.48	23.15 ± 3.67	23.62 ± 3.56	0.359	0.116	0.327
Tumor location				0.645	> 0.999	0.496
Head and Neck	10	21	4			
Body and Tail	11	18	6			
Tumor type				0.629	0.691	0.537
Serous cystic tumor	7	9	2			
Mucinous cystic tumor	3	4	3			
IPMN	4	10	1			
Solid pseudopapillary tumor	5	7	2			
Pancreatic neuroendocrine tumor	2	9	2			
HbA1c (%)	5.29 ± 0.21	5.77 ± 0.32	6.65 ± 0.78	< 0.001	0.002	0.015
CA19-9 (U/ml)	14.08 ± 14.42	21.79 ± 46.83	16.44 ± 12.25	0.466	0.586	0.792
Total bilirubin (μmol/L)	14.67 ± 9.03	11.74 ± 8.54	12.94 ± 5.51	0.219	0.681	0.640
Triglyceride (mmol/L)	1.22 ± 0.56	1.29 ± 0.66	1.34 ± 0.76	0.679	0.514	0.692
Total bile acids (μmol/L)	9.3 ± 19.8	5.7 ± 5.2	5.7 ± 5.4	0.279	0.674	0.824
Pancreatic main duct dilation	4	11	2	0.541	> 0.999	0.709
Tumor volume (cm^3^)	24.28 ± 32.05	22.89 ± 35.61	64.69 ± 74.12	0.882	0.041	0.049
Remnant pancreatic volume (cm^3^)	70.48 ± 14.08	57.44 ± 18.20	37.38 ± 20.40	0.001	< 0.001	< 0.001

P value 1, NGT and pre-DM; P value 2, NGT and NOD; P value 3, pre-DM and NOD.

PBLMT, pancreatic benign and low-grade malignant tumors; NGT, normal glucose tolerance; pre-DM, prediabetes mellitus, NOD, new-onset diabetes; BMI, body mass index; IPMN, intraductal papillary mucinous tumors; CA19-9, carbohydrate antigen 19-9.

### Glucose metabolism in patients with different glucose tolerance statuses.

For β-cell function indices (**Table 2**), HOMA-β was lower in pre-DM (124.40 ± 62.59) and NOD (76.38 ± 34.57) patients than in NGT patients (134.35 ± 35.48, *P* = 0.032 and *P* = 0.001 respectively). In addition, NGT patients had higher levels of IGI (27.44 ± 20.80) than pre-DM (19.09 ± 14.89) and NOD (7.06 ± 8.63) patients (*P* = 0.041 and *P* = 0.021 respectively). Similarly, ISSI-2 was lower in pre-DM (8.91 ± 2.98) and NOD (4.26 ± 2.31) patients than in NGT patients (12.30 ± 4.52, *P* = 0.032 and *P* = 0.001 respectively). HOMA-β, IGI, and ISSI were further decreased in NOD patients compared with pre-DM patients (*P* = 0.015, *P* = 0.001, and *P <* 0.001, respectively). For insulin sensitivity indices, the HOMA-IR and Matsuda index did not differ among the three groups (all *P* > 0.05).

**Table 2:** Glucose metabolism indices of PBLMT patients with different glucose tolerance statuses.

Glucose metabolism indices	NGT (n = 21)	pre-DM (n = 39)	NOD (n = 10)	*P* value 1	*P* value 2	*P* value 3
HOMA-β	134.35 ± 35.48	124.40 ± 62.59	76.38 ± 34.57	0.032	0.001	0.015
HOMA-IR	1.33 ± 0.48	1.56 ± 0.72	1.31 ± 0.42	0.187	0.686	0.561
Matsuda index(pmol/mmol)	137.12 ± 81.09	103.86 ± 63.87	82.42 ± 36.57	0.086	0.071	0.323
IGI (pmol/mmol)	27.44 ± 20.80	19.09 ± 14.89	7.06 ± 8.63	0.041	0.021	0.001
ISSI-2 (pmol/mmol)	12.30 ± 4.52	8.91 ± 2.98	4.26 ± 2.31	0.001	< 0.001	< 0.001

P value 1, NGT and pre-DM; P value 2, NGT and NOD; P value 3, pre-DM and NOD.

PBLMT, pancreatic benign and low-grade malignant tumors; NGT, normal glucose tolerance; pre-DM, prediabetes mellitus, NOD, new-onset diabetes; OGTT, oral glucose tolerance test; HOMA-β, homeostasis model assessment of β-cell function; HOMA-IR, homeostasis model assessment of insulin resistance; IGI, insulinogenic index; ISSI-2, insulin secretion/insulin resistance index.

### Risk factors for PBLMT patients developing dysglycemia

We then classified patients with pre-DM and NOD as dysglycemia groups to assess the risk factors for PBLMT patients developing dysglycemia (**Table 3**). In univariate analysis, age over 60 years old (*P* = 0.014, OR = 6.33, 95% CI: 1.45-27.36), tumor volume more than 24.91 cm^3^ (*P* = 0.033, OR = 4.35, 95% CI: 1.13-16.85), and RPV less than 49.36 cm^3^ (*P* = 0.005, OR = 10.91, 95% CI: 2.06-57.83) were significantly associated with dysglycemia in PBLMT patients. After multivariate analysis, age over 60 years (*P* = 0.049, OR = 5.76, 95% CI: 1.01-32.92) and RPV less than 49.36 cm^3^ (*P* = 0.024, OR = 8.59, 95% CI: 1.34-55.22) were recognized as independent risk factors for dysglycemia.

**Table 3:** Univariate and multivariate analysis of the risk factors for PBLMT patients developing dysglycemia.

Variables	Univariate analysis		Multivariate analysis	
	*P* value	OR (95%CI)	*P* value	OR (95%CI)
Sex	0.151	0.41 (0.12-1.39)	0.194	0.36 (0.08-1.69)
Age (year)				
< 45	Reference		Reference	
45-60	0.104	2.71 (0.81-9.05)	0.291	2.29 (0.49-10.60)
> 60	0.014	6.33 (1.45-27.36)	0.049	5.76 (1.01-32.92)
BMI (kg/m^2^)				
< 18.5	Reference		Reference	
18.5-23.9	0.932	0.92 (0.15-5.74)	0.775	1.40 (0.14-13.98)
> 23.9	0.569	1.75 (0.26-11.99)	0.741	1.50 (0.14-16.42)
Tumor volume (cm^3^)				
< 4.99	Reference		Reference	
4.99-24.91	0.108	2.75 (0.80-9.75)	0.419	1.91 (0.40-9.24)
> 24.91	0.033	4.35 (1.13-16.85)	0.099	4.15 (0.83-20.77)
RPV (cm^3^)				
> 71.74	Reference		Reference	
49.36-71.74	0.091	2.81 (0.85-9.28)	0.114	3.52 (0.74-16.73)
< 49.36	0.005	10.91 (2.06-57.83)	0.024	8.59 (1.34-55.22)

OR, odds ratio; PBLMT, pancreatic benign and low-grade malignant tumors; BMI, body mass index; RPV, Residual pancreatic volume.

### Correlation between RPV and patient characteristics

**Figure 2:**
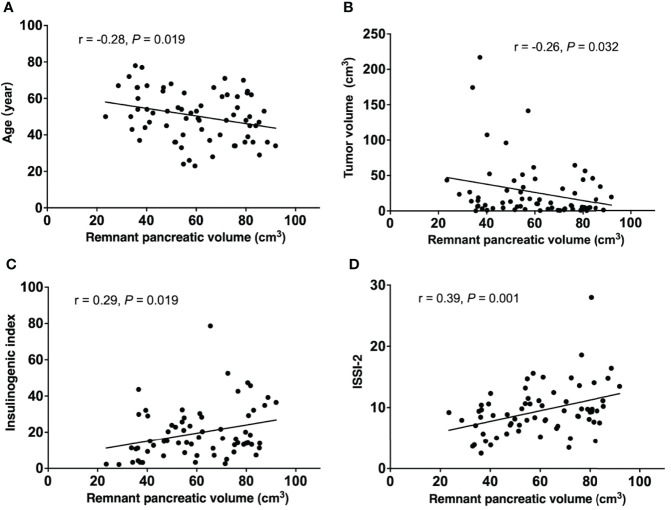
Linear regression analysis between remnant pancreatic volume and **(A)** age, **(B)** tumor volume **(C)** insulinogenic index, and **(D)** ISSI-2 in PBLMT patients. PBLMT, pancreatic benign and low-grade malignant tumors; ISSI-2, insulin secretion/insulin resistance index.

The correlation between RPV and the characteristics of PBLMT patients is shown in **Figure 2**. The analysis of all patients revealed inverse correlations between RPV and both age (r = -0.28, *P* = 0.019, **Figure 2A**) and tumor volume (r = -0.28, *P* = 0.032, **Figure 2B**). Furthermore, positive correlations were found both in IGI (r = 0.29, *P* = 0.019, **Figure 2C**) and ISSI-2 (r = 0.39, *P* = 0.0011, **Figure 2D**).

## Discussion

In the present prospective observational study, 55.7% and 14.3% of PBLMT patients developed pre-DM and NOD, respectively, before pancreatic surgery. Both were higher than the overall prevalence of DM among adult Chinese individuals (35.7% and 10.9%, respectively) (18). When both fasting plasma glucose (FPG) and 2-hour postprandial plasma glucose were used to detect dysglycemia, the prevalence of NOD in PBLMT patients was comparable to that in PNET patients (9.4%) and IPMN patients (6%) in the previous studies (10, 11). However, the prevalence of pre-DM in our study was more than seven times higher than that previously found in PNET patients (7.1%), which was assessed by preoperative FPG measurement only (10). Indeed, more patients with prediabetes could be ignored by FPG alone than by combined OGTT (19, 20). Therefore, we think our study may reflect the actual dysglycemia status of PBLMT patients. Prediabetes is a high-risk state for diabetes development and is associated with an increased risk of kidney disease, diabetic retinopathy, and macrovascular disease (21). Specific risk factors for dysglycemia in PBLMT patients need to be identified.

Changes in pancreas volume have been highlighted as a feature of DM (22, 23). Pancreatic atrophy is a persistent feature in patients with type 1 diabetes, and research has shown that the size and contour of the pancreas are altered in patients with type 2 diabetes (24). In a Mendelian randomization analysis performed by Martin and colleagues, pancreas volume was negatively associated with type 1 diabetes and type 2 diabetes and provided evidence for a causal role of decreased risk of type 2 DM (25). Therefore, it appears that the alteration in pancreas volume could predict DM. Nevertheless, there are still different views on the compensatory capacity of residual pancreatic parenchyma after partial pancreatectomy. King et al. considered that the loss of pancreatic parenchyma after surgical resection did not affect the endocrine function of the pancreas (26). Shirakawa demonstrated that RPV<56% was an independent risk factor for new postoperative diabetes (27). However, the heterogeneity of the included pathologies in these studies and the lack of observational arms might fail to account for the inherent risk of NOD associated with the underlying disease process. Indeed, our study found that the RPV of PBLMT patients had changed before pancreatic surgery, which was reflected in the progressive decrease in RPV in the NGT, pre-DM, and NOD groups.

Loss of pancreatic parenchyma could directly lead to a decrease in various endocrine cells, thus affecting insulin secretion and glucose metabolism (28). OGTT analysis in our study indicated that abnormal glucose metabolism in patients with PBLMT was positively correlated with a decrease in β-cell function. Indices of insulin sensitivity did not differ among the three groups. In addition, RPV in PBLMT patients was positively correlated with IGI and ISSI-2 in linear regression analysis. Thus, we might claim that the reduced RPV and the consequent reduction in adequate β-cell quantity and insulin secretion increased the risk of dysglycemia in PBLMT patients.

In the present study, by evaluating the various risk factors for pre-DM and NOD, RPV was recognized as one of the significant risk factors by multivariate analysis. The primary question arising from this result is what affected the change in RPVs. Tumors in the pancreas, especially those with large tumor volumes, might physically compress the surrounding normal parenchyma resulting in atrophy. A large tumor volume was observed in the NOD group but was not correlated with dysglycemia in PBLMT patients. Similarly, there is still insufficient evidence to suppose that diabetes secondary to pancreatic cancer is due to local effects of tumor infiltration (29). Although RPV shows a negative correlation with tumor volume, our study proposes that dysglycemia might occur only when the tumor causes a decrease in RPV. Another hypothesis is that pancreatic tumors may obstruct the pancreatic ductal system and induce low-grade pancreatitis distal to the lesion with subsequent parenchymal atrophy and endocrine dysfunction. In a randomized clinical study, Tran K et al. revealed that the incidence of new-onset DM was significantly higher in the pancreatic duct obliteration group (30). However, the presence of pancreatic main duct dilation did not show a difference between the groups in our study. This may be because tumors in the body and tail of the pancreas accounted for half of the enrolled patients, and the relationship between the degree of pancreatic duct dilatation and RPV needs to be assessed. In addition, considering the potential immunopathogenesis of diabetes mellitus secondary to pancreatic cancer (29), the immune microenvironment in the context of PBLMT needs further investigation. Age over 60 years old was also an independent risk factor for PBLMT patients developing dysglycemia in the present study. Indeed, RPV negatively correlated with age in PBLMT patients. Research had already found that pancreatic volume decreased with a proportionately high fat content in those over 60 years of age (22). Comprehensive analysis is needed to assess the factors affecting RPV in PBLMT patients.

Clinically, our findings suggest that decisions to treat PBLMT with resection should hinge more on the risk of dysglycemia as well as potential malignancy. Surgery is recommended when the RPV is sufficient. Moreover, these findings advocate that in patients with PBLMT, where partial pancreatectomy is indicated, parenchymal sparing pancreatectomy may benefit from a reduced risk of developing dysglycemia. A nationwide survey showed that in patients with pancreatic body or tail lesions, a significant advantage in preserving endocrine function was found by middle pancreatectomy compared to standard distal pancreatectomy (4).

Our study also had several limitations. Our patient population was from a high-volume pancreatic surgery center, which introduced the possibility of referral bias and may limit the generalizability of our findings to the general population. Moreover, the sample size was further restricted by the single-center study. Serum insulin antibody was needed to exclude Type 1 DM patients.

In conclusion, 70% of PBLMT patients had dysglycemia before pancreatic surgery. Old age and a reduction in RPV were independent risk factors for developing dysglycemia before pancreatic surgery. The decisions to treat PBLMT with surgical resection might hinge more on the risk of dysglycemia as well as potential malignancy.

## Data availability statement

The original contributions presented in the study are included in the article/**Supplementary Material**. Further inquiries can be directed to the corresponding author.

## Ethics statement

The studies involving human participants were reviewed and approved by the West China Hospital of Sichuan University Biomedical Research Ethics Committee (2014 Trail No.37) and conformed to the provisions of the Declaration of Helsinki. Informed consent was acquired from all individual participants and/or guardians included in the study.

## Author contributions

YC designed the study. RW and YL contributed to the interpretation of data and the preparation of the manuscript. JY and JZ performed the data analysis and contributed to the discussion. All authors contributed to the article and approved the submitted version.

## Funding

This study was supported by the Natural Science Foundation of China, No. 82071746; the Key Research and Development Projects in Sichuan Province, No. 2019YFS0043; and the 1·3·5 Project for Disciplines of Excellence–Clinical Research Incubation Project, No. ZY2017302, West China Hospital, Sichuan University.

## Conflict of interest

The authors declare that the research was conducted in the absence of any commercial or financial relationships that could be construed as a potential conflict of interest.

## Publisher’s note

All claims expressed in this article are solely those of the authors and do not necessarily represent those of their affiliated organizations, or those of the publisher, the editors and the reviewers. Any product that may be evaluated in this article, or claim that may be made by its manufacturer, is not guaranteed or endorsed by the publisher.
